# Honokiol protects against diabetic retinal microvascular injury via sirtuin 3-mediated mitochondrial fusion

**DOI:** 10.3389/fphar.2024.1485831

**Published:** 2024-10-22

**Authors:** Jiemei Shi, Min Liu, Jiajie Zhao, Ye Tan, Chunhui Jiang

**Affiliations:** ^1^ Department of Ophthalmology and Vision Science, Eye and ENT Hospital, Fudan University, Shanghai, China; ^2^ Key Laboratory of Myopia and Related Eye Diseases, NHC, Shanghai, China; ^3^ Department of Ophthalmology, Gongli Hospital of Shanghai Pudong New Area, Shanghai, China

**Keywords:** diabetic retinopathy, retinal microvascular endothelial cells, sirt3, honokiol, mitochondria

## Abstract

**Introduction:**

Mitochondrial dysfunction and oxidative stress play important roles in diabetic retinal vascular injuries. Honokiol (HKL) is a small-molecule polyphenol that exhibits antioxidant effects and has a beneficial effect in diabetes. This study aimed to explore the potential ability of HKL to ameliorate vascular injury in diabetic retinopathy (DR) and its possible mechanisms of action.

**Methods:**

The effect of HKL was evaluated in vascular injury in an *in vivo* type 2 diabetic (db/db) mouse model. *In vitro*, retinal microvascular endothelial cells were treated with high glucose (HG) to simulate the pathological diabetic environment. Cell viability, expression of apoptosis-related proteins, cellular reactive oxygen species, mitochondrial membrane potential, and morphological changes in the mitochondria were examined.

**Results:**

The diabetic mice exhibited severe retinal vascular damage, including vascular leakage *in vivo* and capillary endothelial cell apoptosis *in vitro*. HKL reversed the retinal vascular leakage in the diabetic mice. *In vitro*, HKL improved retinal capillary endothelial cell viability, decreased apoptosis, and reversed the HG-induced increased cellular oxidative stress and mitochondrial fragmentation. The sirtuin 3 (SIRT3) inhibitor 3-TYP blocked all the *in vivo* and *in vitro* protective effects of HKL against diabetic retinal vascular leakage and capillary endothelium and eliminated the decrease in oxidative stress levels and reduction of mitochondrial fragmentation.

**Discussion:**

In conclusion, these findings suggest that HKL inhibits vascular injury in DR, which was likely achieved through SIRT3-mediated mitochondrial fusion. This study provides a potential new strategy for the treatment of DR.

## Graphical Abstract

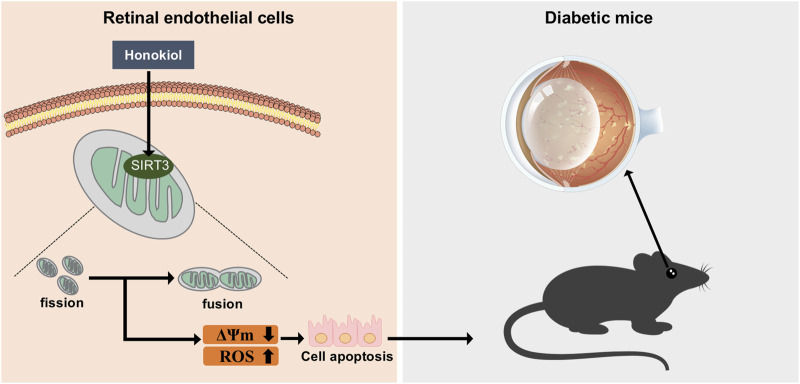

## 1 Introduction

Diabetic retinopathy (DR) is a severe microvascular complication of diabetes mellitus that has become one of the leading causes of vision impairment in adults globally. This has placed a heavy burden on public health systems and negatively affects both the quality of life and the mental health of patients (Cheung et al., 2010). The main pathological changes in the early stage of DR are hyperglycemia-induced damage to retinal cells, mainly affecting the microvascular system, including pericyte loss and endothelial apoptosis, which manifests as vascular leakage (Hammes, 2018). Possible causes of vascular damage have been widely studied, and in the 1960s, hyperglycemia was found to be associated with mitochondrial dysfunction (Brownlee, 2005). Furthermore, compelling evidence supports the pivotal role of mitochondrial dysfunction in the development of diabetic retinal microvascular injury (Wu et al., 2018; Wu and Zou, 2022; Kowluru, 2005).

Honokiol (HKL), 2-(4-hydroxy-3-prop-2-enyl-phenyl)-4-prop-2-enyl-phenol, is a natural bisphenol derived from the magnolia bark. It crosses the blood-brain barrier (BBB) and has diverse pharmacological activities, including anti-inflammatory, antioxidant, anti-tumor, analgesic, and neuroprotective (Fried and Arbiser, 2009; Rauf et al., 2021). HKL is known as an anti-oxidative molecule (Shen et al., 2010; Dikalov et al., 2008). It has traditionally been used as a medicinal compound for the treatment of inflammatory diseases. Because it crosses the BBB and blood-cerebrospinal fluid, HKL exhibits significant bioavailability in neurological tissues with minimal toxicity (Khatoon et al., 2023).

Previous studies have demonstrated that HKL attenuates high glucose (HG)-induced Schwann cell injury and peripheral nerve dysfunction (Hu M. et al., 2023). It also improved renal function in diabetic nephropathy (Rather et al., 2023), ameliorated diabetes-associated cognitive dysfunction (Chang et al., 2023), and protected human umbilical vein endothelial cells against apoptosis under hyperglycemic conditions (Sheu et al., 2008). However, whether HKL alleviates microvascular injury during DR progression and the potential underlying mechanisms are unknown.

HKL has been demonstrated to be a pharmacological activator of sirtuin 3 (SIRT3) (Zhang et al., 2020). SIRT3 is a member of the SIRT family, which is a highly conserved family of nicotinamide adenine dinucleotide (NAD+)-dependent enzymes, consisting of members SIRT1–7 (Wątroba et al., 2017). SIRT3 is located in the mitochondria where it plays a major role in controlling metabolism, function, biogenesis, and dynamics by regulating mitochondrial proteins (Zhang et al., 2020). Recently, SIRT3 downregulation was observed in the heart (Guo et al., 2022), kidneys (Li et al., 2022), and brain (Chang et al., 2023) of diabetic mice and rats.

This protein or gene was also identified in retinal pigment epithelial cells (Huang et al., 2022), umbilical cord blood mesenchymal stem cells (Oh et al., 2019) and umbilical vein endothelial cells (Chen et al., 2021) treated with high-glucose (HG). The reported downregulation of SIRT3 expression in the retina of streptozotocin-induced diabetic rats (Mao et al., 2020) suggested the possible involvement of SIRT3 in DR progression. Therefore, this study aimed to explore the protective effects of HKL against diabetic retinal microvascular injury and its potential involvement in SIRT3 activity.

## 2 Materials and methods

### 2.1 Animal models and experimental groups

All animal care and experimental procedures adhered strictly to the Guidelines for the Care and Use of Laboratory Animals published by the National Institutes of Health in 2011 and were approved by the Eye and ENT Hospital of Fudan University, China (IACUC-DWZX-2021–025). Male BKS wild-type and BKS-db mice (BKS- *Leprem2Cd479/Gpt*, 8-week-old) were purchased from the Jicui Pharmacon Biotechnology Company (Jiangsu, China). The mice were categorized into the following experimental groups and treated as indicated: wt/wt and db/db groups, comprising wild-type and db/db mice, respectively that were fed standard chow for 6 weeks, followed by intraperitoneal injections of phosphate buffered saline (PBS) containing less than 3% dimethyl sulfoxide (DMSO, ST038, Beyotime Biotechnology, China) for two consecutive weeks. And the db/db + HKL (HY-N0003, MCE, United States) group, comprising db/db mice that were fed standard chow for 6 weeks, followed by intraperitoneal injections of HKL at 0.4 mg kg^−1^·day^−1^ (diluted in PBS containing less than 3% DMSO) for two consecutive weeks.

The mice were housed in an animal facility maintained at 23°C ± 2°C and 60%–70% humidity, with free access to food and water, and kept on a 12-h light/dark cycle. The animals were handled in accordance with the guidelines of the Association for Research in Vision and Ophthalmology Statement for the Use of Animals in Ophthalmic and Vision Research.

### 2.2 Cell culture and treatment

Primary rat retinal microvascular endothelial cells (RMECs) (catalog no. RA-6065; Cell Biologics Company, Chicago, IL, United States) were cultured in low-glucose Dulbecco’s modified Eagle’s medium (DMEM, 10,567,014, Invitrogen, United States) supplemented with 10% fetal bovine serum (FBS, 10,099,141, Gibco, Australia), and 1% antibiotic solution (penicillin/streptomycin, 1,514,012, Thermo Fisher, United States). The cells were cultured under conditions of 5% CO_2_ and 37°C. The RMECs from passages 2–6 were used in the experiments. The RMECs were divided into the following groups and treated as indicated: (1) normal glucose (NG) group, cultured in normal DMEM; (2) osmic control (OSM) group, cultured in DMEM medium containing 30 mM mannitol (M108831; Aladdin, Shanghai, China); (3) HG group, cultured in DMEM medium containing 30 mM D-glucose (HY-N0003, MCE, United States); (4) HG + HKL group, cultured in HG DMEM medium containing 30 mM D-glucose (G7021, Sigma-Aldrich, St. Louis, MO, United States) + 10 µM HKL (diluted in PBS containing less than 0.1% DMSO); (5) HG + 3-TYP(HY-108331, MCE, United States) group, cultured in HG DMEM medium containing 30 mM D-glucose +30 µM 3-TYP(3-TYP dissolved in PBS); and (6) HG + 3-TYP + HKL group, pretreated for 2 h with HG DMEM medium containing 30 µM 3-TYP and 30 mM D-glucose, and then 10 µM HKL was added to the medium.

### 2.3 Fundus fluorescein angiography (FFA)

The mice were anesthetized using a mixture of tiletamine hydrochloride and zolazepam (Zoletil, 50 mg/kg) and xylazine hydrochloride (6.25 mg/kg) administered intraperitoneally, and then they received bupivacaine hydrochloride eye drops (Santen, China) for eyeball surface anesthesia. Subsequently, compound tropicamide eye drops (Santen, China) were applied to fully dilate the pupils, and a layer of carbomer gel (Bausch + Lomb Inc., United States) was applied to the corneal surface to protect the cornea. Next, the mice were injected with 10% fluorescein sodium (46,955, Sigma-Aldrich, St. Louis, MO, United States) intraperitoneally and then placed on a special animal holder with the probe aligned to their pupils. A digital fundus camera (OptoProbe Research Ltd., Burnaby, Canada) was used to identify the fundus using fluorescein angiography.

### 2.4 Evans Blue assay

Mice were anesthetized using an intraperitoneal injection of a mixture of Zoletil and xylazine hydrochloride. Evans blue (E2129, Sigma-Aldrich, St. Louis, MO, United States) working solution (45 mg/mL, 0.1 mL/20 g) was injected through the mouse tail vein, allowed a 10-min systemic circulation, and then the eyes were extracted and fixed with 4% paraformaldehyde (PFA, P0099, Beyotime Biotechnology, China) at 35°C for 1 h. The anterior segment, lens, and vitreous humor were removed, and the retinal tissue was carefully peeled off. The retina was carefully cut into four sections and placed flat on a slide. An antifade reagent was applied, and the slide was covered with a coverslip. Finally, images were captured using a confocal microscope (LCM SP-2, Leica Microsystems, Switzerland).

### 2.5 Cell viability assay

Cell viability was assessed using a Cell Counting Kit-8 (CCK-8, C0037, Beyotime, Shanghai, China). Cells were seeded in a 96-well plate, treated with the specified treatments, and then 10 μL CCK-8 solution was added to each well. After incubation at 37°C for 2–4 h, the absorbance values of the reaction solution in each well were measured at 450 nm using a microplate reader.

### 2.6 Calcein-AM/propidium iodide (PI) fluorescence

The calcein-AM/PI double staining method (C2015, Beyotime, Shanghai, China) was used to detect live and dead cells according to the manufacturer’s instructions. Calcein-AM and PI (1 μmol/L each) were added to the wells of a 24-well plate containing cells that had been stimulated with drugs for an appropriate duration. After incubation at 37°C for 30 min in the dark, the cells were examined and photographed using a fluorescence microscope (Axio Observer, ZEISS vision care, Germany).

### 2.7 Determination of reactive oxygen species (ROS)

The intracellular levels of ROS were measured using a fluorescent probe 2′,7′-dichlorodihydrofluorescein diacetate (DCFH-DA) detection kit (S0033, Beyotime, Shanghai, China) according to the manufacturer’s instructions. Briefly, cells were seeded in a 96-well plate and treated under various conditions. Subsequently, DCFH-DA was diluted in serum-free culture medium to a suitable final concentration of 10 μmol/L and added to cover the cells, which were then incubated in the dark at 37°C for 30 min. Then, the cells were washed three times with serum-free culture medium to remove any unloaded probes, and finally, they were examined and photographed using a fluorescence microscope.

### 2.8 Measurement of mitochondrial membrane potential (ΔΨm)

Cells were seeded in 24-well plates, cultured, and exposed to the different predetermined treatments, and then the cell culture medium was removed, followed by gentle washing once with phosphate-buffered saline (PBS, AM9624, Invitrogen, United States). Subsequently, a 1:1 ratio of cell culture medium and JC-1 staining working solution (C2006, Beyotime, Shanghai, China) was added, and the cells were incubated at 37°C for 30 min. Then, the cells were washed twice with JC-1 staining buffer, 1 mL cell culture medium was added to each well, and lastly, the cells were examined using a fluorescence microscope.

### 2.9 Western blotting

Proteins were extracted and lysed using a radioimmunoprecipitation assay lysis buffer (P0013B, Beyotime, Shanghai, China) with ultrasonic treatment on ice. The lysate was then centrifuged at 12,000 × *g* for 10 min at 4°C, the supernatant was collected, and then the protein content was determined using a bicinchoninic acid protein assay (Beyotime, Shanghai, China). The quantified total protein samples were loaded onto various lanes of 10% sodium dodecyl sulfate-polyacrylamide gel electrophoresis gels, and the separated proteins were transferred onto polyvinylidene fluoride membranes.

After blocking, the membranes were incubated with the following primary antibodies overnight in a shaker at 4°C: monoclonal mouse anti-β-actin (1:5,000, A5441, Sigma-Aldrich, St. Louis, MO, United States), monoclonal rabbit anti-SIRT3 (1:1,000, 3,637, Cell Signaling Technology, United States), and monoclonal rabbit anti-optic atrophy 1 (OPA1) (1:1,000, 80,471, Cell Signaling Technology, United States). After washing, the membranes were incubated with horseradish peroxidase-conjugated anti-rabbit and anti-mouse IgG secondary antibodies (1:5,000, 711–035-152 and 715–035-150, Jackson ImmunoResearch Laboratories, United States) for 2 h. The blots were washed and developed using a chemiluminescent reagent (BeyoECL Plus, P0018, Beyotime, Shanghai, China).

### 2.10 Mitochondrial staining and morphological analysis

An appropriate number of cells was seeded in a culture plate and stimulated with predetermined treatments. The cell culture medium was discarded, and MitoTracker working solution (C1035, Beyotime, Shanghai, China) was added, followed by incubation at 37°C for 30 min. The MitoTracker working solution was aspirated and the cells were washed three times with PBS, followed by the addition of fresh culture medium, and live cells were examined using a confocal microscope (LCM SP-2, Leica Microsystems, Switzerland).

In a parallel experiment, cells were fixed with 4% PFA for 30 min, mounted on slides, and stored for later confocal microscopy examination. The ImageJ software program was used to analyze the mitochondrial morphology, and statistical analysis was performed using the structural quantification method proposed by Durand et al. (2019).

### 2.11 Statistical analysis

Statistical analyses were performed using GraphPad Prism software version 8. The data were obtained from at least three independent experiments and are expressed as the means ± standard deviation. Multiple comparisons were performed using a one-way analysis of variance, followed by Bonferroni’s multiple comparison test. Results with a P-value < 0.05 were considered statistically significant.

## 3 Result

### 3.1 HKL reduced retinal vascular leakage in diabetic mice

**FIGURE 1:**
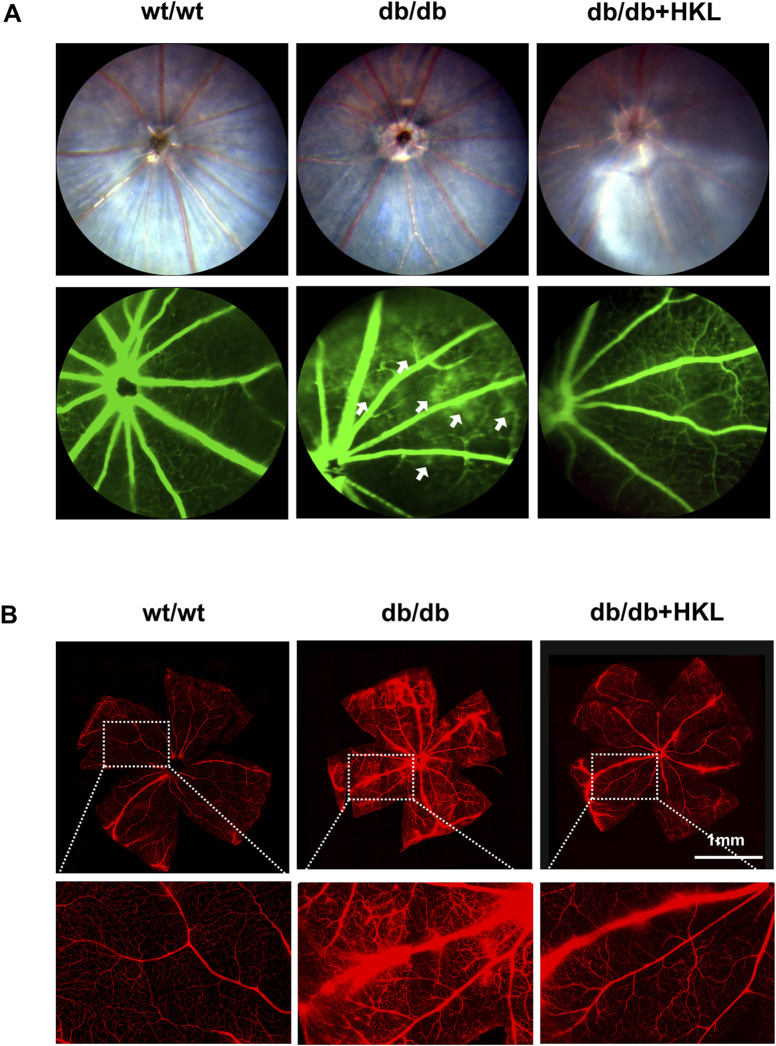
Effects of honokiol (HKL) on diabetic mice. **(A)** Fundus fluorescein angiography showing the effect of HKL on fundus vasculopathy. The white arrows indicate fluorescence leakage points (n = 3). **(B)** Evans Blue staining showing the effect of HKL on the retinal vasculature (scale bars: 1 mm, n = 3). wt/wt: control group; db/db: diabetic group; db/db + HKL: HKL-treated diabetic group.

The FFA imaging analysis of the mice showed that compared to the control group, the diabetic mice exhibited significant points of fluorescence leakage in the late stage, whereas the leakage points were significantly reduced in the HKL-treated diabetic mice (Figure 1A). The examination of the Evans blue-stained retinal vascular system of the mice showed increased permeability in the diabetic group, which was characterized by patchy leakage points. This effect was greatly ameliorated by HKL treatment (Figure 1B).

### 3.2 HKL protected against diabetes-induced RMEC impairment

**FIGURE 2:**
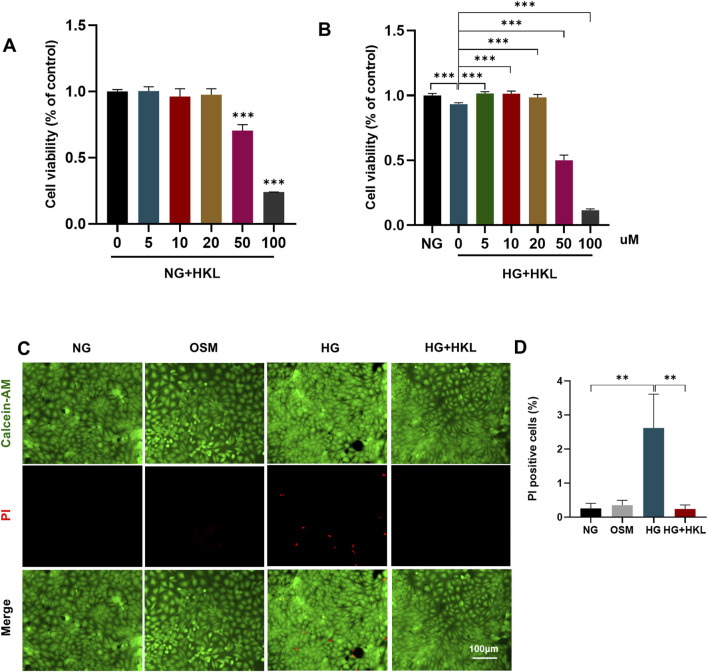
Honokiol (HKL) protects against retinal microvascular endothelial cell (RMEC) Impairment. **(A)** The effect of different HKL concentrations on the viability of RMECs under NG. **(B)** The effect of different HKL concentrations on the viability of RMECs under HG. **(C)** Calcein-AM/propidium iodide (PI) double staining was used to stain dead and live cells. Green: Calcein-AM-labeled live cells, Red: PI-labeled dead cells (scale bars: 100 μm). **(D)** Percentages of PI-positive cells. Values are expressed as means ± SEM, n = 5, ns P > 0.05, *P < 0.05, **P < 0.01, **P < 0.001.

Under normal conditions, HKL levels of 50uM could cause a significant decrease in cell viability (P < 0.001; Figure 2A), possibly due to the toxic effects of high concentrations. Further, the addition of HKL did not exert any significant influence on cellular status or mitochondrial function (Supplementary Figure 1). Our results demonstrated that elevated HG levels decreased cell viability (HG vs. NG, P < 0.0001). Furthermore, HKL effectively reversed the decreased cell viability at all three concentrations (5, 10, and 20 μM; all P < 0.001; Figure 2B). However, there were no significant differences among the three groups (all P > 0.05). Based on these results and similar findings from previous studies, (Qiu et al., 2015; Pillai et al., 2015), 10 µM HKL was selected for subsequent experiments.

Calcein-AM/PI double staining of dead and live cells showed that the proportion of PI-positive cells in the HG and HG + HKL groups was 2.62% and 0.24%, respectively (HG + HKL vs. HG, P = 0.0065). Thus, HKL reversed the HG-induced cell death (Figures 2C, D).

### 3.3 HKL reduced ROS levels and improved mitochondrial function

**FIGURE 3:**
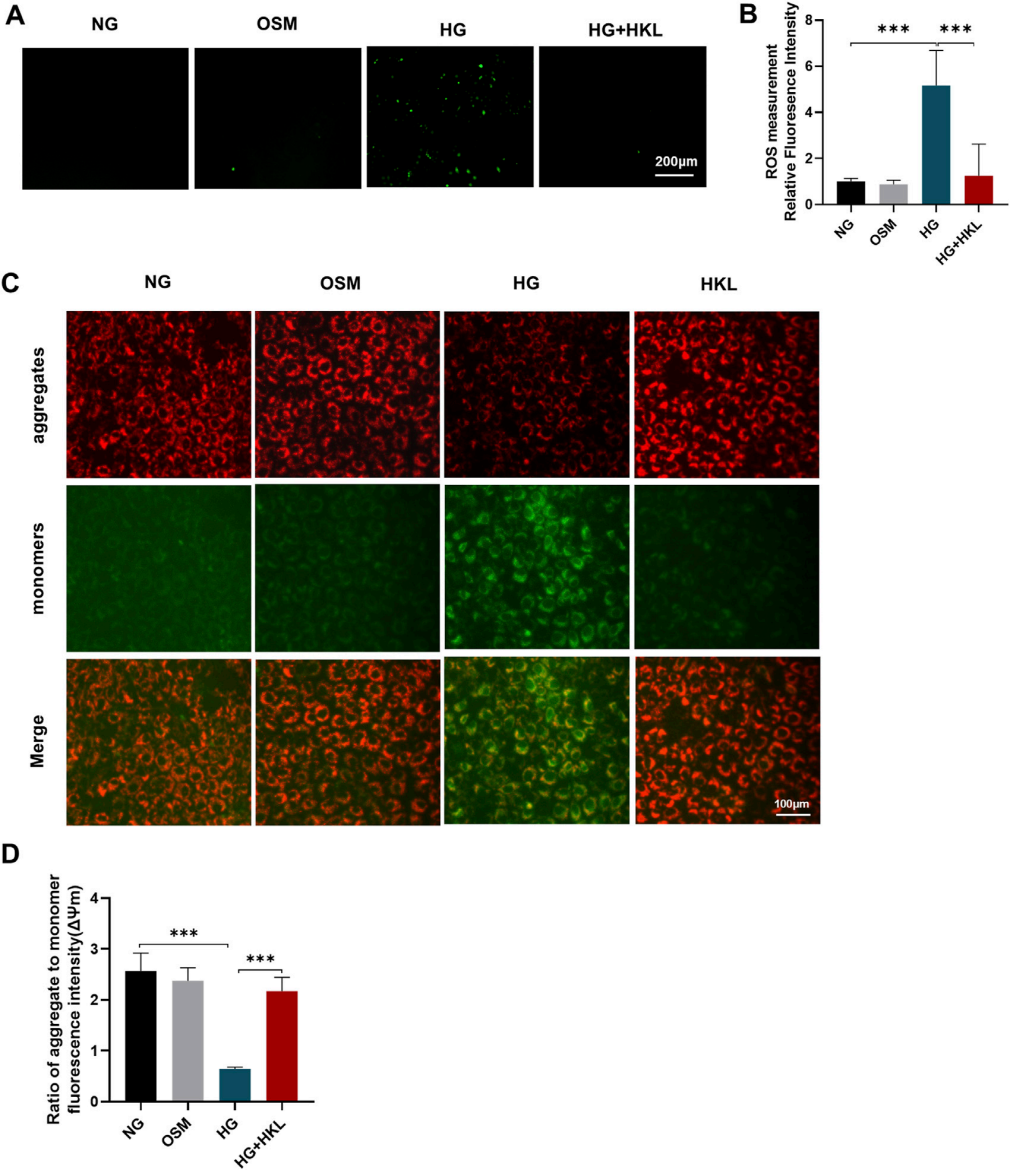
Effect of honokiol (HKL) on oxidative stress and mitochondrial dysfunction. **(A)** Expression levels of reactive oxygen species (ROS) by fluorescence microscopy (scale bars: 200 μm). **(B)** ROS relative fluorescence intensity. **(C)** The mitochondrial membrane potential was measured by JC-1 staining (scale bars: 100 μm). Red: JC-1 aggregates, Green: JC-1 monomers. **(D)** Ratio of aggregate to monomer fluorescence intensity. Values are expressed as means ± SEM, n = 5, ns P > 0.05, *P < 0.05, **P < 0.01, ***P < 0.001.

The results of the DCFH-DA fluorescent probe labeling on examining ROS levels in the cells showed that the HG-induced elevation of oxidative stress levels was effectively reversed by HKL (ROS: HG vs. NG, P = 0.0234; HG + HKL vs. HG, P = 0.0361; Figures 3A, B). Mitochondria are the main source of ROS production, and a decline in the ΔΨm is a sign of mitochondrial damage and early cell apoptosis (Cremers et al., 2018). HG significantly inhibited ΔΨm in cells compared with the control (HG vs. NG, P < 0.0001), and the HKL reversed the decrease in ΔΨm induced by HG (HG + HKL vs. HG, P < 0.0001; Figures 3C, D). These results showed that HKL significantly diminished ROS production and maintained the ΔΨm balance in the HG group.

### 3.4 HKL exerted protective effects on RMECs through SIRT3

**FIGURE 4:**
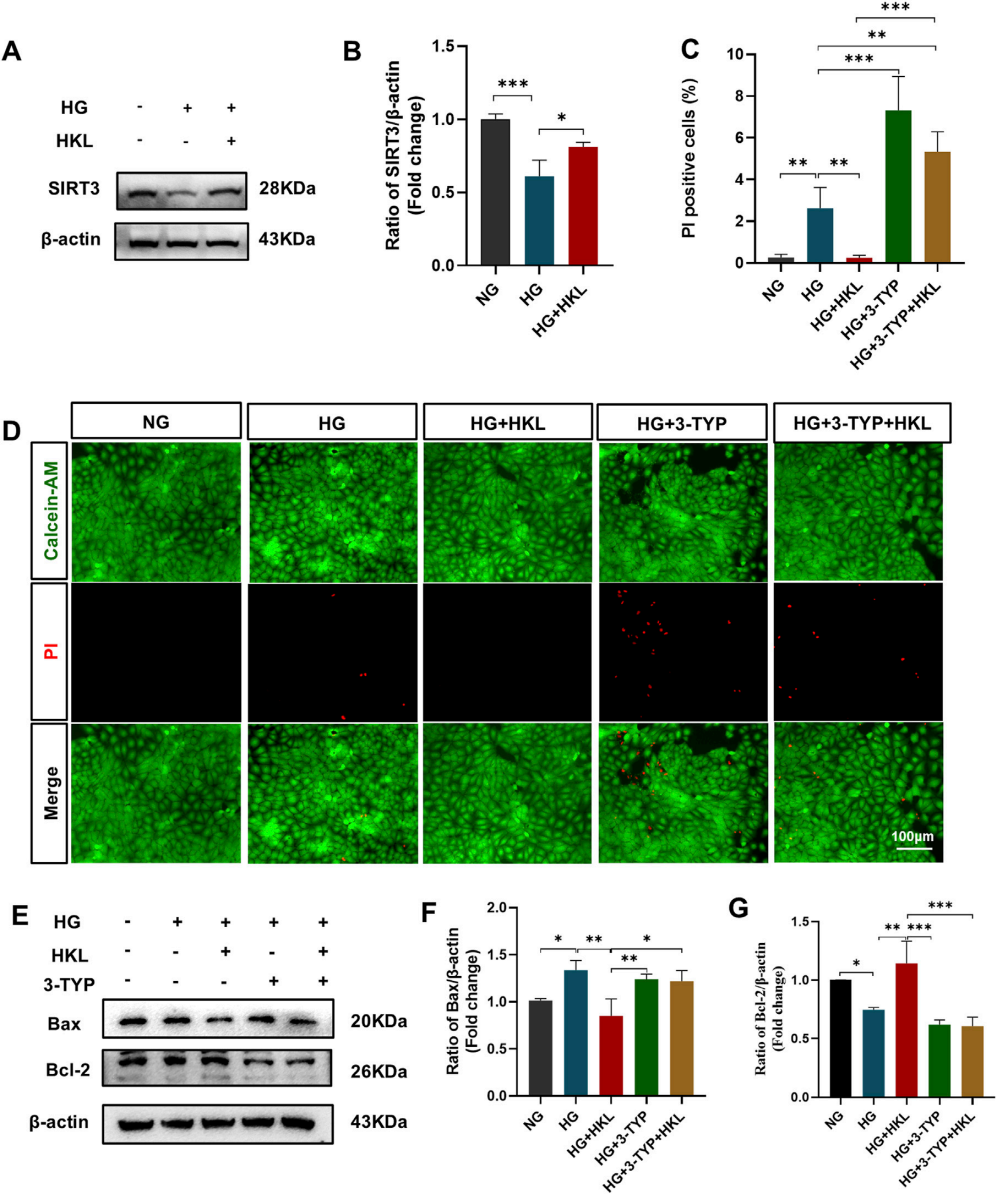
Honokiol (HKL) protects against retinal microvascular endothelial cell (RMEC) impairment through sirtuin 3 (SIRT3). **(A, B)** Expression levels of SIRT3 (n = 3). **(C)** Calcein-AM/propidium iodide (PI) double staining was used to stain dead and live cells. Green: Calcein-AM-labeled live cells, Red: PI-labeled dead cells (scale bars: 100 μm, n = 5). **(D)** Percentage of PI-positive cells. **(E–G)** Expression levels of apoptosis-related proteins (n = 3). Values are expressed as means ± SEM, ns P > 0.05, *P < 0.05, **P < 0.01, ***P < 0.001.

The expression of SIRT3 (HG vs. NG, P = 0.002) was significantly reduced in the HG group; however, it was significantly augmented by HKL (HG + HKL vs. HG, P = 0.0222; Figures 4A, B). The SIRT3 inhibitor 3-TYP blocked the protective effect of HKL in RMECs, where the proportion of PI-positive cells was 5.33% and 0.24% in the HG + 3-TYP + HKL and HG + HKL groups, respectively (HG + 3-TYP + HKL vs. HG + HKL, P < 0.0001; Figures 4C, D). In addition, 3-TYP reversed the decreased expression of the anti-apoptotic protein Bcl2 apoptosis regulator (Bcl2; HG + 3-TYP + HKL vs. HG + HKL, P = 0.0003), whereas that of the pro-apoptotic protein Bcl2 associated X apoptosis regulator was increased (HG + 3-TYP + HKL vs. HG + HKL; P = 0.0139, Figures 4E, F).

**FIGURE 5:**
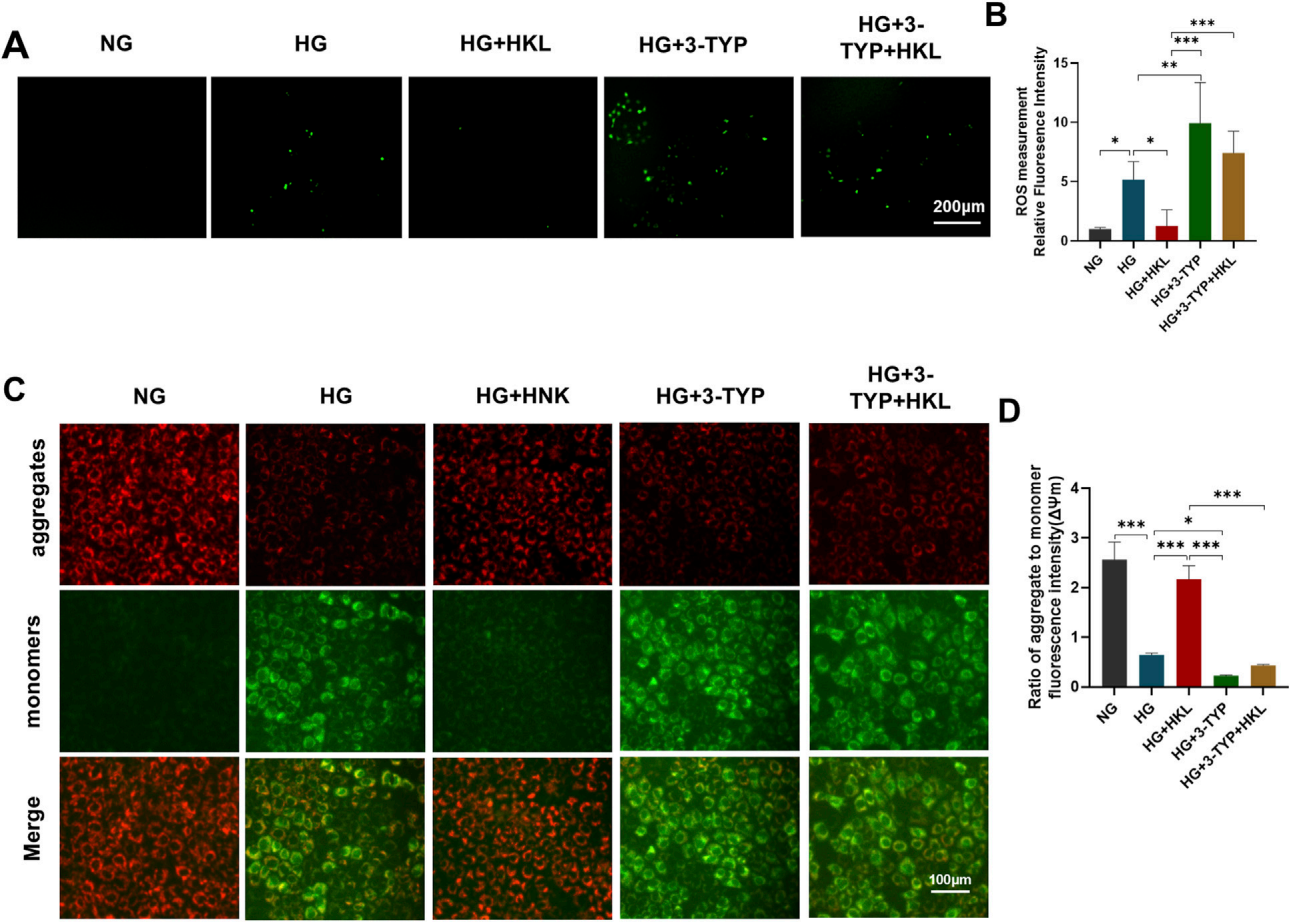
Honokiol (HKL) protects against oxidative stress and mitochondrial dysfunction through sirtuin 3 (SIRT3). **(A)** Expression levels of reactive oxygen species (ROS) by fluorescence microscopy (scale bars: 200 μm). **(B)** ROS relative fluorescence intensity. **(C)** The mitochondrial membrane potential was measured by JC-1 staining (scale bars: 100 μm). Red: JC-1 aggregates, Green: JC-1 monomers. **(D)** Ratio of aggregate to monomer fluorescence intensity.

Treatment with the SIRT3 inhibitor 3-TYP abolished the protective effects of HKL on cellular ROS levels (HG + 3-TYP + HKL vs. HG + HKL, P = 0.0006; Figures 5A, B) and the ΔΨm (HG + 3-TYP + HKL vs. HG + HKL; P < 0.0001; Figures 5C, D).

### 3.5 HKL protected RMECs through SIRT3-mediated mitochondrial fusion

**FIGURE 6:**
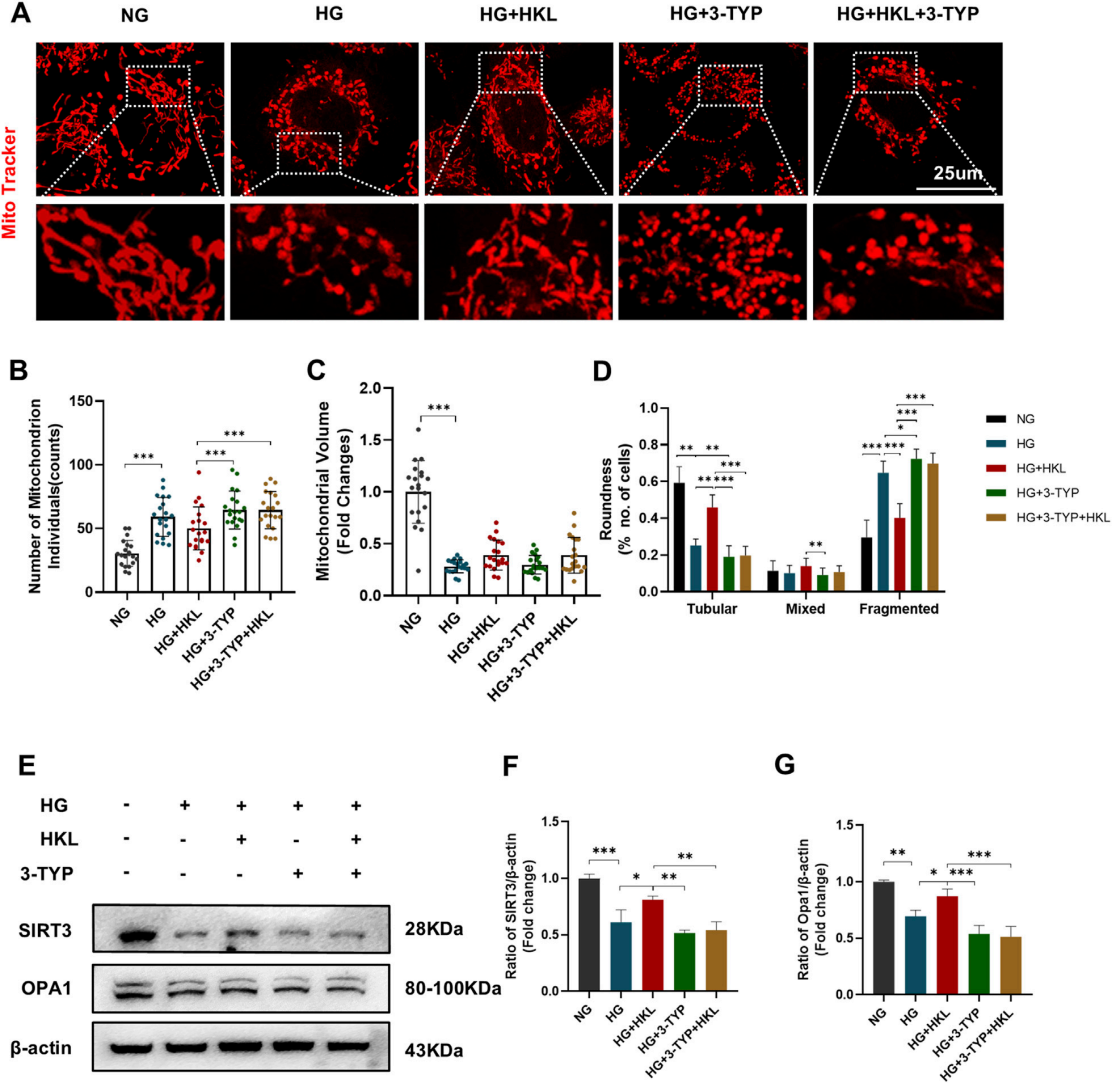
Honokiol (HKL) improves mitochondrial fusion through sirtuin 3 (SIRT3). **(A)** Mitochondrial morphology in living cells by confocal microscopy (scale bars: 25 μm). **(B)** Mitochondrial numbers. **(C)** Mitochondrial volumes. **(D)** Mitochondrial roundness. At least 20 mitochondria were included in 3 independent experiments. **(E–G)** Expression levels of SIRT3 and OPA1 (n = 3). Values are expressed as means ± SEM, ns P > 0.05, *P < 0.05, **P < 0.01, ***P < 0.001.

MitoTracker fluorescent probes were used to label the mitochondria (Figure 6A). Figures 6B–D shows that compared to the levels in the control group, HG increased the mitochondrial number and decreased the volume in the HG group (HG vs. NG, both P < 0.0001). Meanwhile, the tubular mitochondria were decreased, whereas the fragmented mitochondria were increased (HG vs. NG, both P < 0.0001).

Furthermore, the proportion of the tubular mitochondria increased after treatment with HKL, whereas that of the fragmented mitochondria decreased (HG + HKL vs. HG, both P < 0.0001). Intervention with the SIRT3 inhibitor 3-TYP blocked the protective effect of HKL on mitochondrial fragmentation (tubular and fragmented: HG + 3-TYP + HKL vs. HG + HKL, both P < 0.0001).

Our examination of the mitochondrial dynamics-related protein OPA1 showed that its expression level decreased in the HG group, and this was accompanied by a decrease in SIRT3 expression (OPA1 and SIRT3: HG vs. NG, P = 0.0013 and 0.0002, respectively). Furthermore, treatment with HKL increased the expression of OPA1, which was blocked by the SIRT3 inhibitor (OPA1: HG + HKL vs. HG, P = 0.0464; HG + 3-TYP + HKL vs. HG + HKL, P = 0 .0004, Figures 6E–G).

## 4 Discussion

The current study demonstrated that HKL mitigated HG-induced retinal vascular impairment, and this protective effect might be attributable to SIRT3–OPA1 signaling-mediated improvement in mitochondrial fusion. To our knowledge, this is the first study to demonstrate the retinal protective actions and related underlying mechanisms of action of HKL against DR. In the present study, we found that HKL protected diabetic mice against retinal vascular leakage.

*In vitro* treatment of RMECs with HKL reversed the decrease in cell viability, apoptosis, ΔΨm, and oxidative stress caused by HG. These findings suggest a potential therapeutic value of HKL against vascular injury in DR. Previous studies have demonstrated that HKL showed an anti-inflammatory effect in palmitic acid-inducted endothelial dysfunction (Qiu et al., 2015). In addition, HKL reduced HG-induced Schwann cells injury by activating the AMPK/SIRT1/PGC-1α pathway and enhancing mitochondrial function (Hu M. et al., 2023). Further, another study found that HKL improve chronic cerebral hypoperfusion-induced neurological damage by inhibiting astrocyte A1 polarization via regulating SIRT3-STAT3 axis (Hu Y. et al., 2023). Interestingly, in recent years, HKL has been shown to pharmacologically activate SIRT3 (Zhang et al., 2020), Pillai et al. have demonstrated that HKL directly bound to SIRT3 to enhance its activity in cardiomyocytes, elevating its expression level by approximately two-fold (Pillai et al., 2015). Wang et al. (2018) and Ye et al. (2019) reported that HKL upregulated SIRT3 protein expression even in a dose-dependent manner. Moreover, numerous studies have confirmed that its significant actions in various diseases are mediated through SIRT3 signaling. These diseases include cardiovascular, cerebrovascular, (Zheng et al., 2018; Liu et al., 2022; Chi et al., 2020), and neurodegenerative (Ramesh et al., 2018) diseases; tumors (Luo et al., 2017); infections (Kim et al., 2019; Yu et al., 2022); and pulmonary fibrosis (Cheresh et al., 2021). Our evaluation of the effects of HKL on SIRT3 expression demonstrated that its protein levels in RMEC increased after HKL treatment. 3-TYP is a most widely used selective SIRT3 inhibitor (Zhang et al., 2020) with high selective SIRT3 inhibition (SIRT3 IC_50_ = 16 nM) (Galli et al., 2012). Here, we used 3-TYP to verify whether the protective effect of HKL was affected by SIRT3 signaling. The results showed that the protective effect of HKL was antagonized by the SIRT3 inhibitor 3-TYP, which further demonstrated that HKL exerted its protective effects through SIRT3.

SIRT3, which is expressed in the mitochondria, is an important regulator of mitochondrial homeostasis (Zhang et al., 2020) and various physiological and pathophysiological processes in conjunction with other SIRT family members (Wątroba et al., 2017). Three SIRTs (SIRT3–5) are located in the mitochondria, and (Yang et al., 2016) found that SIRT3 interacts with most substrates (at least 84 mitochondrial proteins). This observation strongly indicates that SIRT3 might be the most critical SIRT mitochondrial regulator. Several studies have reported the importance of SIRT3 in cardiovascular diseases and diabetes-related complications (Dikalova et al., 2020; Li et al., 2022; Dikalova et al., 2017).

SIRT3 expression is significantly decreased in the arterioles of patients with hypertension, and transgenic mice overexpressing SIRT3 exhibited a reversal of hypertension, endothelial dysfunction, and vascular oxidative stress (Dikalova et al., 2020). Extensive vascular endothelial dysfunction often occurs in early sepsis, and SIRT3 plays an important role in its pathogenesis (Yu et al., 2022). SIRT3 is also involved in the destruction of the endothelial cell barrier function (Wu et al., 2020). SIRT3 expression was found to decrease in the myocardium of diabetic mice, and knockout of SIRT3 aggravated cardiomyocyte necrosis and cardiac dysfunction (Song et al., 2021).

Although SIRT3 plays an important role in cardiovascular diseases and other diabetes-related complications, few studies have investigated its role in DR. Gao et al. (2016) reported that SIRT3 overexpression attenuates hyperglycemic injury through deacetylation and activation of manganese superoxide dismutase in bovine retinal capillary endothelial cells. Our results showed that the protective effects of HKL against mitochondrial dysfunction and ROS increase under HG were mediated through SIRT3. These findings highlighted the importance of SIRT3 in DR.

Mitochondria are the energy centers of cells and are involved in regulating cellular biological processes, metabolism, and apoptosis (Sorrentino et al., 2018). Mitochondria are highly dynamic organelles that individually express various morphologies controlled by opposing processes of fusion and fission (Chan, 2020). By regulating the dynamics of fusion and fission, the mitochondria facilitate cellular adaptation to changing energy requirements, thereby maintaining normal cell function (Yu and Pekkurnaz, 2018). Unbalanced mitochondrial dynamics lead to excessive mitochondrial ROS production and imbalanced oxidative homeostasis (Willems et al., 2015).

To elucidate the potential mechanisms underlying the involvement of SIRT3 in the protective actions of HKL, we examined and quantified the mitochondria in live cells. The results showed that HG increased mitochondrial fission in RMECs, which is consistent with the findings of a previous study (Kim et al., 2020).

Furthermore, HKL treatment significantly decreased the proportion of fragmented mitochondria. These results suggest that the actions of HKL mediated through SIRT3 enhanced mitochondrial fusion. We further examined the mitochondrial dynamics-related fusion protein OPA1, which mediates the fusion of the inner mitochondrial membrane (Cipolat et al., 2004). We found that HKL likely acting through SIRT3 reversed the HG-induced mitochondrial fragmentation, and the expression levels of OPA1 and SIRT3 were closely related, indicating that SIRT3 potentially regulates mitochondrial fusion via OPA1. However, the direct or indirect interactions between OPA1 and SIRT3 require further study.

There are some limitations to our study that warrants further investigation. First, experiments *in vivo* were insufficient as we did not comprehensively evaluate the effects of HKL in the *db/db* mice. The number of animals in the study was restricted, and a clinically applicable dose and administration mode need to be further explored. Second, the SIRT3 activity need be further evaluated and interactions between SIRT3 and OPA1 remains unclear; whether there are other molecules involved in these actions requires further study. Moreover, we focused on observing the effects of HKL on retinal microvascular abnormalities in this study. As HKL has demonstrated possible neuroprotective effects in several studies (Hu M. et al., 2023; Bibi et al., 2023; Zhou et al., 2023; Hu Y. et al., 2023), further studies of its efficacy on retinal neurologic function are required.

## 5 Conclusion

Our findings suggest that HKL attenuates HG-induced vascular injury in DR, possibly through SIRT3-mediated mitochondria fusion. These results suggest that HKL may have potent therapeutic usefulness in the treatment of microvascular damage during the early stages of DR.

## Data Availability

The raw data supporting the conclusions of this article will be made available by the authors, without undue reservation.
