# Supplementary figures and images for: Honokiol protects against diabetic retinal microvascular injury via sirtuin 3-mediated mitochondrial fusion
Source: Front Pharmacol. 2024 Oct 22;15:1485831. doi: 10.3389/fphar.2024.1485831 (PMC11574205; doi:10.3389/fphar.2024.1485831)

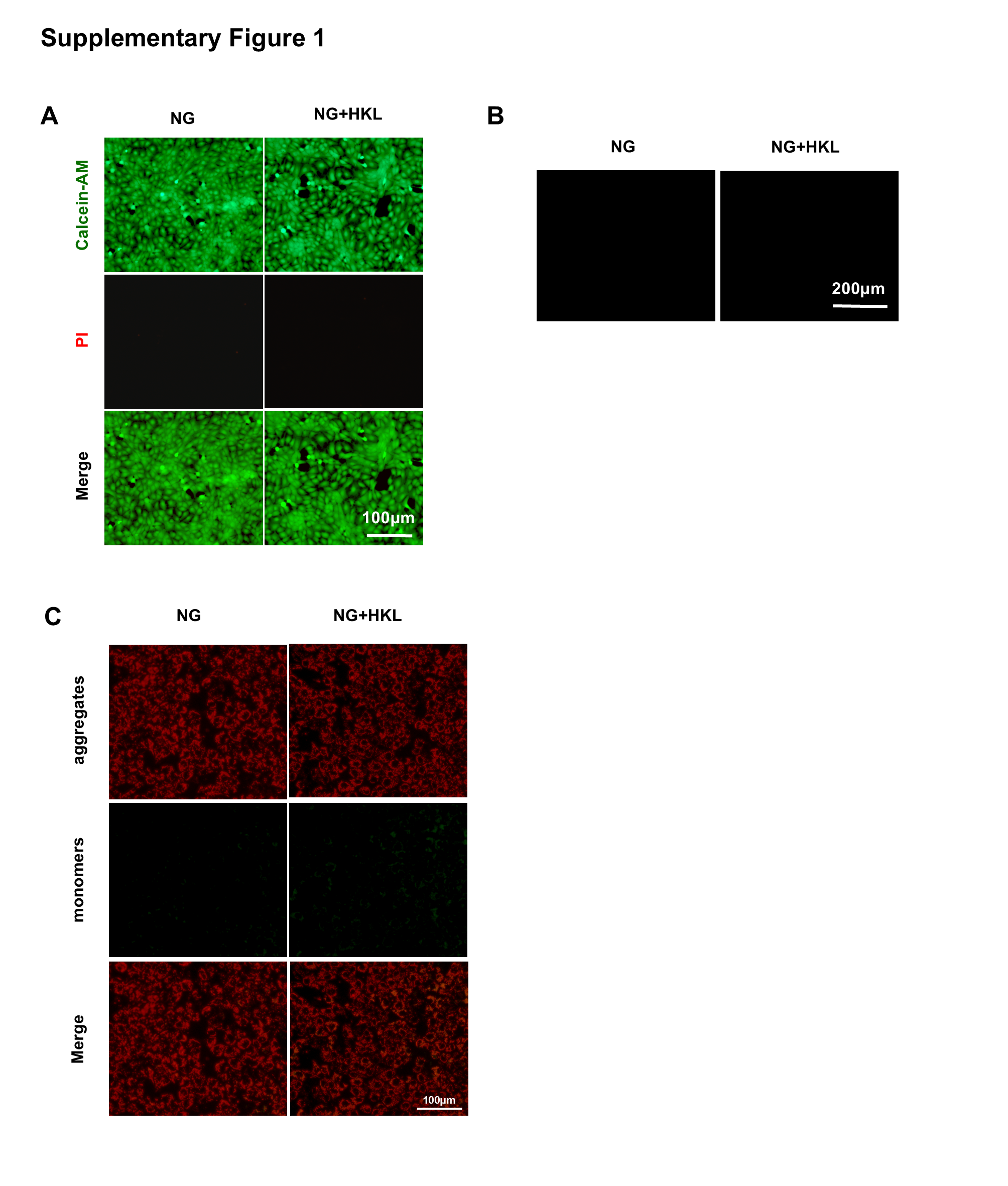

Supplement: Supplementary file 1 [file Image1.TIF]
